# Role of ABC Proteins in Secondary Metabolism and Immune (=Defensive) Response in Seaweeds

**DOI:** 10.3390/cells12182259

**Published:** 2023-09-12

**Authors:** Leonardo T. Salgado, Louisi S. Oliveira, Juliana Echevarria-Lima, Vanessa M. Reis, Daniela B. Sudatti, Fabiano L. Thompson, Renato C. Pereira

**Affiliations:** 1 Diretoria de Pesquisas, Instituto de Pesquisas Jardim Botânico do Rio de Janeiro, Rio de Janeiro 22460-030, Brazil; vanessamouradosreis@gmail.co; 2 Departamento de Biotecnologia Marinha, Instituto de Estudos do Mar Almirante Paulo Moreira—IEAPM, Arraial do Cabo 28930-000, RJ, Brazil; louisi.oliveira@marinha.mil.br; 3 Laboratório de Imunologia Celular e Aplicada, Departamento de Imunologia, Instituto de Microbiologia Paulo Góes, Universidade Federal do Rio de Janeiro, Rio de Janeiro 21941-590, Brazil; juechevarria@micro.ufrj.br; 4 Departamento de Biologia Marinha, Instituto de Biologia, Universidade Federal do Rio de Janeiro, Rio de Janeiro 21941-590, Brazil; dbsudatti@id.uff.br; 5 Departamento de Biologia Marinha, Instituto de Biologia, SAGE-COPPE, Universidade Federal do Rio de Janeiro, Rio de Janeiro 21941-590, Brazil; fabianothompson1@gmail.com

**Keywords:** chemical defense, fouling, herbivory

## Abstract

*Laurencia* seaweed species synthesize a broad range of secondary metabolites, mainly terpenes (e.g., elatol), exhibiting diverse ecological roles, such as defense against fouling and herbivores. Recently, an intricate cellular machinery was described concerning terpenes biosynthetic pathways, storage inside *corps en cerise* (CC), and regulated exocytosis in these species. But for seaweeds in general, the proteins involved in transmembrane transport of secondary metabolites remain unknown. Assays with Rhodamine-123 and cyclosporine A (CSA) revealed the presence of ABC transporters in CC membrane of *Laurencia dendroidea*. In vivo incubation assays with CSA resulted in CC morphological changes, reduced intracellular elatol concentrations, and increased biofouling cover on the seaweed surface. Cultivation assays in the presence of a marine pathogenic bacteria induced the expression of ABC proteins belonging to the subfamilies ABCB, ABCD, ABCF, and ABCG. The latter subfamily is known to be associated with the transport of plant terpenes. Our results shed new light on the role of ABC proteins in key mechanisms of the defensive system in seaweeds against fouling and herbivory.

## 1. Introduction

*Laurencia* is a well-known chemically defended seaweed genus whose many species produce mainly terpenes and acetogenins, which act in defense against microbes, herbivores, competitors, and epiphytes [1]. The halogenated sesquiterpene elatol is one of the major specialized metabolites common in this genus and occurs in variable intrathallus concentrations [2]. In red seaweeds, diverse intracellular structures were previously characterized as sites for the storage and/or biosynthesis of defensive chemicals, such as gland cells [3] and mevalonosomes [4]. Halogenated metabolites in *L. dendroidea* are stored inside specialized vesicles called *corps en cerise* (CC) [5,6], and their main metabolite may be the sesquiterpene elatol [2]. These substances are released to cell surfaces through regulated vesicle trafficking [7,8] activated by epiphytic bacteria whose growth are, thereafter, chemically inhibited [9].

Recent advances to the knowledge of defensive strategies in *L. dendroidea* include the characterization of a large array of genes involved in the biosynthesis of terpenes [10], and the recognition of diverse molecular mechanisms activated in response to epiphytic marine pathogenic bacteria [11]. Nevertheless, although *L. dendroidea* has been developed as a model study for seaweed chemical defense [6,7,8,9], the transport systems involved in the accumulation of specialized metabolites inside CC remain unknown.

In plants, vacuoles are not only the end-storage cellular compartment for defense compounds, they also host biosynthetic intermediates and the final steps of biosynthetic pathways. For example, the transport of defensive compounds is promoted by three types of transporters: ATP-binding cassette (ABC) protein, multidrug and toxin extrusion protein (MATE), and nitrate/peptide family transporters (NPFs) [12]. The ABC superfamily is one of the largest proteins in all organism, comprising membrane-bound transporters and soluble proteins that are grouped in eight subfamilies—from ABCA to ABCH [13]. ABC transporters play diverse roles in plant cell events, acting as importers and exporters, and also play a leading in the growth, nutrition, and resistance to stress [14].

Moreover, ABC transporters are considered biotechnological targets, since they are involved in the compartmentalization of specialized metabolites (secondary metabolites) to avoid autotoxicity, which improves cell growth in microbial cultures for the heterologous biosynthesis of plant valuable compounds [12,13,14]. In addition, microbes harboring not only biosynthetic genes but also genes for certain ABC transporters could exudate the specialized metabolites in the culture medium, increasing the recovery efficiency [15]. In that sense, expanding the knowledge about ABC proteins would help in setting up heterologous systems for the biosynthesis of biotechnologically relevant seaweeds specialized metabolites.

According to a transcriptome-based study, ABC transporter genes are widespread among seaweed species [16]. Other studies, based on gene expression, suggest that ABC transporters may be involved in seaweeds resistance to the effects of heavy metal [17] and desiccation [18]. Nonetheless, the role of ABC proteins in seaweed secondary metabolites transport and their association with the chemical defense mechanism remain largely unknown. Aiming to elucidate the roles of ABC proteins in seaweeds, this article investigated the intracellular localization of ABC transporters in *L. dendroidea*, as well as their role in the transport and accumulation of secondary metabolites, and the consequent chemical defense mechanism against biofouling.

## 2. Materials and Methods

### 2.1. Obtaining and Maintaining Clones of L. dendroidea

Clones obtained from *L. dendroidea* specimens collected in the intertidal zone at Rasa Beach (Búzios, Rio de Janeiro State, Brazil) were cultivated in seawater enriched with Von Stosch (VS). Clones were re-excised and replicated in culture media, renewed weekly, to obtain a unialgal culture, i.e., free of micro- and macroalgae, and germanium dioxide (1 mg L^−1^) was used to suppress diatom growth when necessary. The culture conditions were the following: temperature: 20 ± 2 °C, salinity: 32 ± 1, irradiance: 40 ± 5 µmol photons.m^−2^.s^−1^, and 14 h light/10 h dark. These culture conditions were used for all experiments unless otherwise stated.

### 2.2. Intracellular Localization of ABC Proteins

The presence of ABC proteins on the membrane of *L. dendroidea* organelles was investigated by incubating trichoblasts with the fluorescent dye Rhodamin-123 (Rho 123) [19] and with the inhibitor of ABC activity, Cyclosporin A (CSA; obtained from Novartis, São Paulo, Brazil) [20]. Trichoblasts are hyaline uniserial and branched filaments grown over seaweed thallus apex, and they were chosen for this experiment due to their high liability, rapid growth, and plasticity to environmental factors and to the absence of auto-fluorescence (from chlorophyll), which interferes with fluorescent dye detection. The concentrations were chosen based on the classical literature on the study of chemical ABC modulation. Four CSA concentrations were used (200, 1000, 2000, and 5000 ng·mL^−1^). However, at higher concentrations (2000 and 5000 ng·mL^−1^), we observed cell death. We verified cell integrity by analyzing nucleus and chloroplast integrity through fluorescence microscopy and using DAPI for DNA labelling and chlorophyll autofluorescence to observe chloroplasts. In addition to CSA, we also evaluated the effect of verapamil (VP) on ABC modulation (initially at 5 and 10 μm). However, even at these low VP concentrations, cells integrity was compromised; thus, VP assays were discontinued. The assays with CSA were set as follows: Assay 1: trichoblasts were incubated with Rho 123 (200 ng·mL^−1^) for 30 min; Assay 2: seaweed fragments were incubated with Rho 123 (200 ng·mL^−1^) for 60 min; Assay 3: trichoblasts were incubated with both Rho 123 (200 ng·mL^−1^) and CSA (1000 ng·mL^−1^) for 60 min. Following this, cells were washed with culture medium and maintained alive at 4 °C until observed at fluorescence optical microscope (Olympus BX 50, 100× objective lens, N.A. 1.3, oil). Images were acquired with a CCD camera Cool Snap-Pro Color RS Photometrics coupled to a computer.

### 2.3. Morphology of corps en cerise (CC), Elatol Content, and Chemical Defense

To evaluate the effects of ABC inhibition on the morphology of CC, the main secondary metabolites storage organelle, and on elatol content, *L. dendroidea* specimens were cultivated with sterile seawater for 10 days and submitted to daily CSA incubation times of 6 h. The CSA concentrations used were control: 0 ng·mL^−1^; low: CSA—200 ng·mL^−1^; high: CSA—1000 ng·mL^−1^. The same experiment setting was used to evaluate the effects of ABC inhibition on antifouling defense, but clones were cultured in nonsterile seawater.

The area of CC (n = 100) in trichoblast cells was analyzed as a function of CSA concentration. Cells were observed with an Olympus BX 50 microscope (100× objective lens, N.A. 1.3, oil) and the differential interferential contrast (DIC) images were acquired with a CCD camera Cool Snap-Pro Color RS Photometrics coupled to a computer. The area of CC was analyzed and measured with ImageJ 1.46r software [21]. Elatol concentration was quantified in hexanic extracts of *L. dendroidea* trichoblasts (N = 5 per treatment) by using a gas chromatograph (Crompack CP 900×) coupled to an electron capture detector (GC-ECD) and based on analytical curves obtained from five standard solutions of purified elatol [2].

To evaluate the effects of ABC inhibition on antifouling defense, the relative surface area of *L. dendroidea* biofouled thalli was evaluated as a function of CSA concentration. Thalli surface images (N = 40) were acquired with an Olympus ZX microscope equipped with a digital Olympus SC30 Color Camera coupled to a computer. The surface area of biofouled thalli was analyzed and measured with ImageJ software [21]. Samples were also processed for scanning electron microscopy, fixed in 2.5% glutaraldehyde and 4% formaldehyde (diluted in sodium cacodilate buffer (0.1 M, pH 7.4). After post-fixation in 1% osmium tetroxide for 1 h, the samples were dehydrated in crescent series of ethanol and dried by CO_2_ critical point method. Representative images of biofouled thalli area from each treatment were obtained with a Zeiss EVO 40 scanning electron microscope.

### 2.4. Expression Level of ABC Protein Candidate Genes

Clones obtained from *L. dendroidea* specimens collected at Castelhanos beach (Anchieta, Espírito Santo State, Brazil) were treated with antibiotics cocktail (100 µg·mL^−1^ ampicillin, 120 µg·mL^−1^ streptomycin, and 60 µg·mL^−1^ gentamicin) and maintained in sterile seawater with germanium dioxide (1 mg·L^−1^) and 50% Provasoli solution (sterile-enriched seawater—SESW) for 2 days before the experiment. The culture and experimental conditions were the following: temperature: 22 ± 1 °C, salinity: 32 ± 1, irradiance: 80 ± 5 µmol photons·m^−2^·s^−1^, and 14 h light/10 h dark. The bacteria *Vibrio madracius,* isolated from the coral *Madracis decactis* [22], was grown at 30 °C in sterile marine broth to the OD_600_ 0.8, corresponding to 10^8^ CFU·mL^−1^, and precipitated for 5 min at 3.000 rpm (Centrifuge 5415R, Eppendorf, Hamburg, Germany). The supernatant was discarded, and the pellet was resuspended in sterile seawater. *Vibrio madracius* is associated with bleached coral and induces the upregulation of genes related to defense responses in *L. dendroidea*, including the biosynthesis of terpenes [11,22].

Control tubes were set up with 250 mg of *L. dendroidea* and 40 mL of SESW (n = 3), and inoculated tubes contained 250 mg of *L. dendroidea*, 40 mL of SESW, and *V*. *madracius* at 10^7^ CFU mL^−1^ (n = 3). After 24, 48, and 72 h, control and inoculated *L. dendroidea* specimens were frozen and ground in liquid nitrogen. Total RNA was extracted using the TRIzol protocol (Invitrogen, Carlsbad, CA, USA), cDNA libraries were prepared using the TruSeq stranded mRNA LT Sample Preparation kit Illumina (Sand Diego, CA, USA), and paired-end sequencing (2 × 250 bp) was performed on a MiSeq (Illumina).

Sequences were assembled using Trinity [23], and sequences larger than 199 bp were used in the downstream analysis. To identify the transcripts coding for ABC proteins, we prospected the transcriptome of *L*. *dendroidea* using hidden Markov models available at PFAM and the HMMER 3.0 software [24]. The ABC protein candidate genes were annotated through a BLAST search against the NCBI-nr and Uniprot databases (e-value < 10^−5^). Sequences from each sample coding for ABC protein were mapped against the assembled reads using Bowtie 2 [25], and statistically relevant differentially expressed genes between the control and the inoculated samples were identified using the edgeR software (version 3.42.4) package associated with Fisher’s exact test and Bonferroni correction for multiple tests (considering corrected *p*-value ≤ 0.001 and logFC ≥ 2.0) [26]. The functional annotation and classification of candidate genes into ABC protein subfamilies was confirmed through SmartBLAST, and the best hits were aligned with *L. dendroidea* candidate genes.

### 2.5. Statistical Analysis

Most statistical analyses were performed using ANOVA with a post hoc analysis (Tukey HSD) to resolve differences among levels of significant factors (*p* < 0.05). Other analyses were performed with PERMANOVA (D1 Euclidean distance) with the PRIMER version 6 with PERMANOVA+. Pairwise comparisons were used to resolve differences among levels of significant factors (*p* < 0.05).

## 3. Results

**Figure 1:**
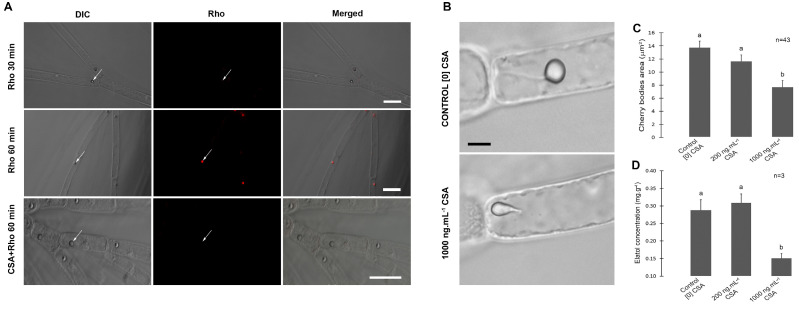
Localization of ABC proteins and the effect of ABC inhibition on cell structure and on specialized (secondary) metabolism. (**A**) *L. dendroidea* trichoblast cells incubated with Rhodamine-123 in the presence and absence of Cyclosporin A. The trichoblast cells seen in the first row were incubated with Rho 123 for 30 min. No dye fluorescence was observed inside the *corps en cerise* (CC). No dye fluorescence was observed inside CC (white arrows) in trichoblast cells incubated with Rho 123 for 30 min. In the second row (after 60 min incubation), detection of Rho 123 was restricted to the lumen of CC, indicating specific transport activity across its membrane. The third row shows trichoblast cells simultaneously incubated with Rho 123 and Cyclosporin A, where Rho 123 accumulation inside CC was inhibited by Cyclosporin A (CSA). Differential interferential contrast (DIC) images are shown in the first column, fluorescence images are shown in the second column, and merged images are shown in the third column. Bars = 30 μm. (**B**) Inhibition of ABC proteins by Cyclosporin A altered the morphology of CC and reduced elatol concentration in trichoblast cells (DIC images). The upper image shows a trichoblast cell and the CC with typical morphology, from control treatment. The below image shows CC with altered morphology in trichoblast cells incubated with CSA (1.000 ng·mL^−1^). Bars = 4 µm. (**C**) Morphometric measurements of CC in trichoblast cells cultured in the presence (200 or 1000 ng·mL^−1^) and absence of CSA (control). (**D**) Elatol concentration in trichoblasts cultured in the presence (200 or 1.000 ng·mL^−1^) and absence of CSA (control). For both graphs, different letters indicate significant differences between treatments (*p* < 0.05).

After incubation with the fluorescent dye Rhodamin-123 (Rho 123) and with the inhibitor of ABC activity, Cyclosporin A (CSA), *L. dendroidea* trichoblast cells showed no fluorescent dye compartmentation after incubation with Rho 123 for 30 min (Figure 1A, and Supplementary materials). However, Rho 123 fluorescence was observed exclusively inside the CC (Figure 1A) after 60 min of incubation. The simultaneous incubation of trichoblast cells with Rho 123 and CSA for 60 min resulted in no fluorescence inside CC, revealing that the fluorescent dye compartmentation was interrupted (Figure 1A).

**Figure 2:**
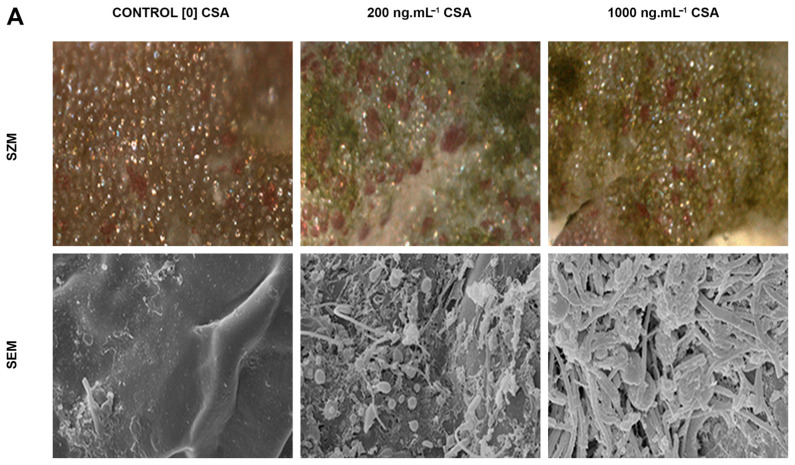

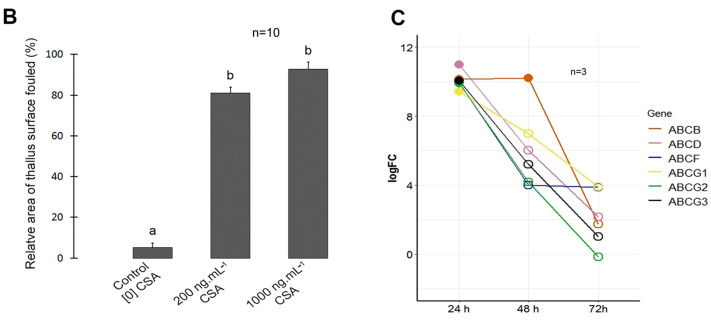
Effects of ABC inhibition on chemical defense against fouling and differential ABC genes expression induced by pathogenic bacteria. (**A**) Inhibition of ABC proteins by Cyclosporin A impaired *L. dendroidea* chemical defense against biofouling. Stereomicroscopy (first row) and scanning electron microscopy (second row) images of thallus surface. Note the drastic increase on biofilm (initial biofouling) coverage over the algal thallus surface in CSA-treated samples. Bars = 150 μm and 7 μm in upper and lower rows, respectively. (**B**) Relative surface area of biofouled thallus as a function of CSA concentration. Different letters indicate significant differences between treatments (*p* < 0.05). (**C**) Differentially expressed genes coding for ABC proteins in *L. dendroidea* 24, 48, and 72 h after inoculation with *Vibrio madracius*. Data represent logFC values considering “inoculated” versus “control” samples. Open circles indicate values of logFC that were not statistically significant (*p* value > 0.001; logFC < |2.0|). ABCG1, ABCG2, and ABCG3 represent three different genes coding for proteins belonging to the ABCG subfamily.

After incubation, we also observed morphological alterations by fluorescence in CC of trichoblast cells incubated with CSA (Figure 1B, and Supplementary materials). The area of CC decreased significantly in cells treated with 1.000 ng·mL^−1^ CSA, when compared to the respective control and the 200 ng·mL^−1^ treatment (Figure 1C and Supplementary materials). Elatol concentration reduced in extracts of trichoblast cells under high CSA treatment when compared to control and low CSA treatment (Figure 1D and Supplementary materials). Thalli surface images revealed that the relative surface area of *L. dendroidea* covered by biofouling increased from ~5% in control samples to ~80% in low CSA and ~90% in high CSA treatments (Figure 2A,B and Supplementary materials).

**Figure 3:**
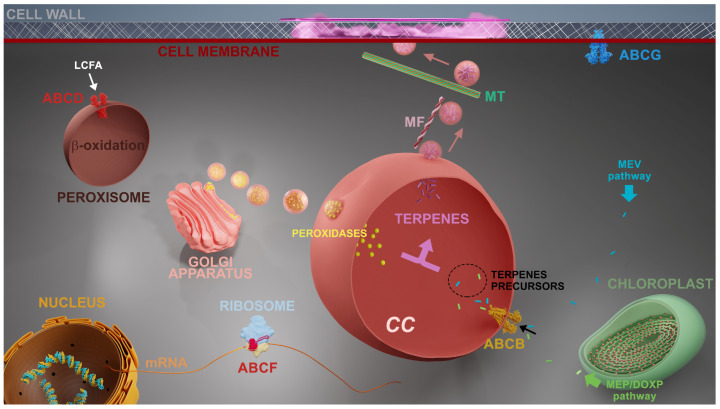
Hypothetical model representing cellular processes elicited in *L. dendroidea* in response to microbes. The first steps of terpenoid biosynthesis occur through mevalonate (MVA) or methylerythritol phosphate (MEP) pathways, taking place in cytoplasm and chloroplasts, respectively. *Corps en cerise* (CC) development depends on the Golgi secretory pathway. ABCB transporters are responsible for importing terpenoid precursors to the lumen of CC. ABCD transporters import long-chain fatty acids to peroxisomes where β oxidation occurs, providing alternative sources of energy for defensive metabolism. ABCF proteins participate in stress response acting on protein synthesis and ribosome assembly. ABCG is involved in the exudation of specialized metabolites across the cell membrane. Vesicles budding from CC [6,7,8,9] are transported through microfilaments (red) until the cell periphery and positioned in regions where exocytosis will occur through microtubules (green) [6], thus inhibiting biofouling [9].

Genes coding for the following subfamilies of ABC proteins were upregulated in *L. dendroidea* 24 h after the inoculation by marine pathogenic bacteria: ABCB, ABCD, ABCF, and ABCG. Three different ABCG genes were upregulated 24 h after bacterial inoculation, named ABCG1, ABCG2, and ABCG3 (Supplementary material). The overexpression of the gene coding for the ABCB transporter was also observed 48 h post-inoculation (Figure 2C). The cytological and molecular evidence presented here were combined with previous knowledge to propose a cellular model regarding the chemical defense mechanisms in *L. dendroidea* (Figure 3).

## 4. Discussion

The ability of seaweeds to store secondary metabolites in specialized vesicles provides protection against cell autotoxicity [27] and allows the regulated release of defensive compounds in response to stimulus or induction—fouling colonization [9]. Fouling has a number of negative effects on seaweeds, including the reduction of growth [28] and reproduction [29], increased drag and, consequently, tissue loss during storms [30], or increased susceptibility to consumers that are attracted to seaweeds possessing fouling organisms [31]. Furthermore, the mechanism by which chemicals are transported to the stalk surface is an essential aspect of understanding surface-mediated interactions [32].

The presence of ABC transporters in *corps en cerise* (CC) membrane of *L. dendroidea* was demonstrated in this study by the compartmentation of Rho 123 in the lumen of this storage vesicle. Accordingly, this compartmentation ceased after the addition of CSA, which is known to impair the activity of ABC transporters [20]. Experimental approaches using Rho 123 and CSA to locate ABC proteins are often applied in plants and, while providing indirect evidence, are quite efficient. The presence of ABC in *L. dendroidea* CC membrane was reiterated due to compromised development of the organelle under the influence of CSA maintained in culture medium. In this sense, the reduced size of CC of cells submitted to higher CSA concentrations indicates that ABC transporters are responsible for the transmembrane transport and accumulation of elatol inside CC, since this organelle is responsible for the storage of such compound [6] and presumably other secondary metabolites. Acting in this context of transport means that it also acts to prevent the known autotoxic action of secondary metabolites of *L. dendroidea* [27].

Moreover, the concentration of elatol in *L. dendroidea* trichoblasts reduced after the addition of high concentrations of CSA, which suggests that ABC transporters could also be (indirectly) involved in the biosynthesis of elatol by acting on the compartmentation of biosynthetic intermediates. Most of the seaweed secondary metabolites are derived from isoprenoids, comprising terpenes, steroids, carotenoids, and others, which are derived from the mevalonic acid pathway (MVA) or from the 2C-methyl-D-erythritol 4-phosphate pathway—MEP [33]. Traditionally, the MVA pathway occurs in the cytoplasm and mitochondria, and the MEP pathway is localized in chloroplasts [4], but the intracellular localization of isoprenoid biosynthesis and storage is poorly understood [34]. The cellular localization of the biosynthetic steps of secondary metabolites has been little explored for seaweeds [4], despite this being an essential aspect for understanding the defensive system. Therefore, knowing precursors and enzymes, and where they occur inside cells, is an essential aspect of understanding the defense system against natural enemies. For instance, vesicle-like intracellular structures, considered to be the precursors of CC, were shown to contain biosynthetic intermediates for terpenoid biosynthesis through the mevalonate pathway in *L. dendroidea* [10]. Consistently, the inhibition of ABC transporters resulted in lower defense amounts against biofouling, as observed by the progressive increase in thallus area biofouled following the increase on CSA concentration.

Furthermore, the presence of a pathogenic marine bacteria in *L. dendroidea* induced the overexpression of genes belonging to four ABC subfamilies: ABCB (MDR), ABCD (PMP), ABCF, and ABCG (PDR). Recent studies suggested a role for ABCB transporters on monoterpene compartmentation and toxicity tolerance in lavender plants [35], and on the import of the alkaloid berberine in the plant species *Coptis japonica* [36]. As such, we hypothesize that ABCB transporters could be also involved in the accumulation of defensive chemicals inside the CC in *L. dendroidea,* but further studies will be necessary to investigate the specific role of ABCB transporters in seaweeds. Evidence that genes encoding members of the ABCD subfamily are twice as abundant in red algae species, Rhodophyta [16], as in plants, seems to support our hypothesis, even though we do not yet know their exact biological functions and/or ecological roles. In plants, these peroxisome-localized transporters are important for long-chain fatty acid metabolism [37].

Accordingly, the oxidation of fatty acids was among the upregulated pathways verified in *L. dendroidea* as a response to manipulated infestation by pathogenic bacteria, possibly providing alternative sources of energy during biotic stress [11]. The ABCF subfamily comprises soluble proteins with unknown functions, even in plants. Studies in yeast and humans have indicated an indirect role of ABCF protein in the regulation of stress response factors, by acting on protein synthesis and ribosome assembly [16]. We suppose that ABCF could be involved in orchestrating biotic stress response in *L. dendroidea*, but specific studies in this topic are required in the future. Finally, ABCG proteins are recognized transporters of terpenoid compounds with antimicrobial activities towards plant surfaces [15,38]_._ These proteins could be relevant for the exudation of defense compounds in *L. dendroidea*, in addition to the well-established and known vesicle trafficking process in this red seaweed [7]. Considering the high molecular diversity and chemical characteristics of secondary metabolites of *L. dendroidea*, it is expected that more than one strategy may be deployed for intracellular transport and exudation of defensive compounds.

In conclusion, the cellular and molecular evidence presented here suggest that ABC transporters play multiple roles in seaweeds’ chemical defense metabolisms, possibly acting on secondary metabolites’ accumulation in specialized vesicles (CC), participating in stress-response signaling and energy metabolism, and promoting the delivery of defensive compounds at pathogens, biofouling contact areas, and other natural but deleterious enemies. Therefore, this study enhances the comprehension of the complex and dynamic mechanisms involved in seaweed chemical defense. Following a biotechnological perspective, this article presents valuable information for the future heterologous biosynthesis of seaweed secondary metabolites, since important advances have been made in the context of genes involved in terpenoid biosynthesis of these chemicals in *L. dendroidea* [10,39]. Even so, the proposed roles for ABC proteins in seaweed should be also investigated through analysis of ABC protein mutants or overexpression and silencing of ABC proteins through seaweed metabolic engineering. Finally, the results presented here demonstrate that the chemical defense mechanisms in seaweeds and land plants are more similar than known so far, despite 400 million years of distinct and independent evolutionary histories.

## Data Availability

Not applicable.

## Supplementary Materials

The following supporting information can be downloaded at: https://www.mdpi.com/article/10.3390/cells12182259/s1, Figure S1. Multiple alignment of LdABCB (translated sequence) and five best hit protein sequences (SmartBLAST). Alignment columns with gaps are colored grey. The red color indicates highly conserved columns and blue indicates less conserved ones; Figure S2. Multiple alignment of LdABCD (translated sequence) and five best hit protein sequences (SmartBLAST). Alignment columns with gaps are colored grey. The red color indicates highly conserved columns and blue indicates less conserved ones; Figure S3. Multiple alignment of LdABCF (translated sequence) and five best hit protein sequences (SmartBLAST). Alignment columns with gaps are colored grey. The red color indicates highly conserved columns and blue indicates less conserved ones; Figure S4: Multiple alignment of LdABCG1 (translated sequence) and five best hit protein sequences (SmartBLAST). Alignment columns with gaps are colored grey. The red color indicates highly conserved columns and blue indicates less conserved ones; Figure S5: Multiple alignment of LdABCG2 (translated sequence) and five best hit protein sequences (SmartBLAST). Alignment columns with gaps are colored grey. The red color indicates highly conserved columns and blue indicates less conserved ones; Figure S6: Multiple alignment of LdABCG3 (translated sequence) and five best hit protein sequences (SmartBLAST). Alignment columns with gaps are colored grey. The red color indicates highly conserved columns and blue indicates less conserved ones; Table S1: Statistical analysis of CC morphometry; Table S2: Statistical analysis of elatol quantification in trichoblasts; Table S3: Statistical analysis of fouling coverage over algae thalli; Table S4: Statistical analysis of ABC gene expression; Table S5: Best hits for *L. dendroidea* gene annotated as ABCB protein (LdABCB) through SmartBLAST; Table S6: Best hits for *L. dendroidea* gene annotated as ABCD protein (LdABCD) through SmartBLAST; Table S7: Best hits for *L. dendroidea* gene annotated as ABCF protein (LdABCF) through SmartBLAST; Table S8: Best hits for *L. dendroidea* gene annotated as ABCG protein (LdABCG1) through SmartBLAST; Table S9: Best hits for *L. dendroidea* gene annotated as ABCG protein (LdABCG2) through SmartBLAST; Table S10: Best hits for *L. dendroidea* gene annotated as ABCG protein (LdABCG3) through SmartBLAST; Table S11. Results of the incubation assay about the effect of CSA on area of the CC; Table S12: Results of the incubation assay about the effect of CSA on elatol content; Table S13: Results of the incubation assay about the indirect effect of CSA on fouling.

## References

[1] Harizani M., Ioannou E., Roussis V. (2016). The *Laurencia* paradox: An endless source of chemodiversity. Prog. Chem. Org. Nat. Prod..

[2] Sudatti D.B., Rodrigues S.V., Pereira R.C. (2006). Quantitative GC-ECD analysis of halogenated metabolites: Determination of surface and within-thallus elatol of *Laurencia obtusa*. J. Chem. Ecol..

[3] Paul N.A., Cole L., De Nys R., Steinberg P.D. (2006). Ultrastructure of the gland cells of the red alga *Asparagopsis armata* (Bonnemaisoniaceae). J. Phycol..

[4] Paradas W.C., Crespo T.M., Salgado L.T., Andrade L.R., Soares A.R., Hellio C., Paranhos R.R., Hill L.J., Souza G.M., Kelecom A.G.A.C. (2015). Mevalonosomes: Specific vacuoles containing the mevalonate pathway in *Plocamium brasiliense* cortical cells (Rhodophyta). J. Phycol..

[5] Young D.N., Howard B.M., Fenical W. (1980). Subcellular localization of brominated secondary metabolites in the red alga *Laurencia snyderae*. J. Phycol..

[6] Salgado L.T., Viana N.B., Andrade L.R., Leal R.N., Da Gama B.A.P., Pereira R.C., Amado Filho G.M. (2008). Intra-cellular storage, transport and exocytosis of halogenated compounds in marine red alga *Laurencia obtusa*. J. Struct. Biol..

[7] Reis V.M., Oliveira L.S., Passos R.M.F., Viana N.B., Mermelstein C., Sant’Anna C., Pereira R.C., Paradas W.C., Thompson F.L., Amado-Filho G.M. (2018). Traffic of secondary metabolites to cell surface in the red alga *Laurencia dendroidea* depends on a two-step transport by the cytoskeleton. PLoS ONE.

[8] Sudatti D.B., Rodrigues S.V., Coutinho R., Da Gama B.A.P., Salgado L.T., Amado-Filho G.M., Pereira R.C. (2008). Transport and defensive role of elatol at the surface of the red seaweed *Laurencia obtusa* (Ceramiales, Rhodophyta). J. Phycol..

[9] Paradas W.C., Salgado L.T., Sudatti D.B., Crapez M.A., Fujii M.T., Coutinho R., Pereira R.C., Amado Filho G.M. (2010). Induction of halogenated vesicle transport in cells of the red seaweed *Laurencia obtusa*. Biofouling.

[10] Oliveira L.S., Tschoeje D.A., Oliveira A.S., Hill L.J., Paradas W.C., Salgado L.T., Thompson C.C., Pereira R.C., Thompson F.L. (2015). New Insights on the terpenome of the red seaweed *Laurencia dendroidea* (Florideophyceae, Rhodophyta). Mar. Drugs.

[11] Oliveira L.S., Tschoeke D.A., Lopes A.C.R.M., Sudatii D.B., Meirelles P.M., Thompson C.C., Pereira R.C., Thompson F.L. (2017). Molecular mechanisms for microbe recognition and defense by the red seaweed *Laurencia dendroidea*. mSphere.

[12] Brito F.R., Martinoia E. (2018). The vacuolar transportome of plant specialized metabolites. Plant Cell Physiol..

[13] Verrier P.J., Bird D., Burla B., Dassa E., Forestier C., Geisler M., Klein M., Kolukisaoglu U., Lee Y., Martinoia E. (2008). Plant ABC proteins—A unified nomenclature and updated inventory. Trends Plant Sci..

[14] Hwang J.U., Song W.-Y., Hong D., Ko D., Yamaoka Y., Jang S., Yim S., Lee E., Khare D., Kim K. (2016). Plant ABC transporters enable many unique aspects of a terrestrial plant’s lifestyle. Mol. Plant..

[15] Shitan N. (2016). Secondary metabolites in plants: Transport and self-tolerance mechanisms. Biosci. Biotechnol. Biochem..

[16] Lane T.S., Rempe C.S., Davitt J., Station M.E., Peng Y., Soltis D.E., Melkonian M., Deyholos M., Leebens-Mack C.M., Rothfels C.J. (2016). Diversity of ABC transporter genes across the plant kingdom and their potential utility in biotechnology. BMC Biotechnol..

[17] Ritter A., Dittami S.M., Goultquer S., Correa J.A., Boyen C., Potin P., Tonon T. (2014). Transcriptomic and metabolomic analysis of copper stress acclimation in *Ectocarpus siliculosus* highlights signaling and tolerance mechanisms in brown algae. BMC Plant Biol..

[18] Fierro C., Lópes-Cristoffanini C., Meynard A., Lovazzano C., Castañeda F., Guajardo E., Conttreras-Porcia L. (2017). Expression profile of desiccation tolerance factors in intertidal seaweed species during the tidal cycle. Planta.

[19] Forster S., Thumser A.E., Hood N., Plant N. (2012). Characterization of Rhodamine-123 as a tracer dye for use in in vitro drug transport assays. PLoS ONE.

[20] Qadir M., O’Loughlin K.L., Fricke S.M., Williamson N.A., Greco W.R., Minderman H., Baer M.R. (2005). Cyclosporin A is a broad-spectrum multidrug resistance modulator. Clin. Cancer Res..

[21] Schindelin J., Rueden C.T., Hiner M.C., Eliceiri K.W. (2015). The ImageJ ecosystem: An open platform for biomedical image analysis. Mol. Rep. Dev..

[22] Moreira A.P.B., Duytschaever G., Tonon L.A.C., Dias G.M., Mesquita M., Cnockaert M., Francini-Filho R.B., De Vos P., Thompson C.C., Thompson F.L. (2014). *Vibrio madracius* sp. nov. isolated from *Madracis decactis* (Scleractinia) in St Peter & St Paul Archipelago, Mid-Atlantic Ridge, Brazil. Curr. Microbiol..

[23] Grabherr M.G., Haas B.J., Yassour M., Levin J.Z., Thomspn D.A., Amit I., Adiconis X., Fan L., Raychowdhury R., Zeng Q. (2011). Full-length transcriptome assembly from RNA-Seq data without a reference genome. Nat. Biotechnol..

[24] Finn R.D., Clements J., Eddy S.R. (2011). HMMER web server: Interactive sequence similarity searching. Nucleic Acids Res..

[25] Langmead B., Salzberg S.L. (2012). Fast gapped-read alignment with Bowtie 2. Nat. Methods.

[26] Robinson M.D., McCarthy D.J., Smyth G.K. (2010). EdgeR: A Bioconductor package for differential expression analysis of digital gene expression data. Bioinformatics.

[27] Sudatti D.B., Duarte H.M., Soares A.R., Salgado L.T., Pereira R.C. (2020). New ecological role of seaweed secondary metabolites as autotoxic and allelopathic. Front. Plant Sci..

[28] Meichssner R., Stegmann N., Cosin A.-S., Sachs D., Bressan M., Marx H., Krost P., Schulz R. (2020). Control of fouling in the aquaculture of *Fucus vesiculosus* and *Fucus serratus* by regular desiccation. J. Appl. Phycol..

[29] Saier B., Chapman A.S. (2004). Crusts of the alien bryozoan *Membranipora membranacea* can negatively impact spore output from native kelps (*Laminaria longicruris*). Bot. Mar..

[30] Dixon J., Schroeter S.C., Kastendiek J. (1981). Effects of the encrusting bryozoan, *Membranipora membranacea*, on the loss of blades and fronds by the giant kelp, *Macrocystis pyrifera* (Laminariales). J. Phycol..

[31] Da Gama B.A.P., Santos R.P.A., Pereira R.C. (2008). The effect of epibionts on the susceptibility of the red seaweed *Cryptonemia seminervis* to herbivory and fouling. Biofouling.

[32] De Nys R., Dworjanyn S.A., Steinberg P.D. (1998). A new method for determining surface concentrations of marine natural products on seaweeds. Mar. Ecol. Prog. Ser..

[33] Maschek J.A., Baker B.J., Amsler C.D. (2008). The chemistry of algal secondary metabolism. Algal Chemical Ecology.

[34] Lohr M., Schwender J., Polle J.E.W. (2012). Isoprenoid biosynthesis in eukaryotic phototrophs: A spotlight on algae. Plant Sci..

[35] Demissie Z.A., Tarnowycz M., Adai A.M., Sarjer L. (2019). A lavender ABC transporter confers resistance to monoterpene toxicity in yeast. Planta.

[36] Otani M., Shitan N., Sakai K., Martinoia E., Sato F., Yazaki K. (2005). Characterization of vacuolar transport of the endogenous alkaloid berberine in *Coptis japonica*. Plant Physiol..

[37] Charton L., Plett A., Linka N. (2019). Plant peroxisomal solute transporter proteins. J. Integr. Plant Biol..

[38] Lefèvre F., Boutry M. (2018). Towards identification of the substrates of ATP-binding cassette transporters. Plant Physiol..

[39] Oliveira L.S., Gregoracci G.B., Silva G.G.Z., Salgado L.T., Amado Filho G.M., Ferreira M.A., Pereira R.C., Thompson F.L. (2012). Transcriptomic analysis of the red seaweed *Laurencia dendroidea* (Florideophyceae, Rhodophyta) and its microbiome. BMC Genom..

