# Supplementary material for: Role of ABC Proteins in Secondary Metabolism and Immune (=Defensive) Response in Seaweeds
Source: Cells. 2023 Sep 12;12(18):2259. doi: 10.3390/cells12182259 (PMC10526433; doi:10.3390/cells12182259)

Table S1. Statistical analysis of CC morphometry.

**Data summary**					
			**Samples**		
	**Control**	**200**	**1000**	**Total**	
N	43	43	43	129	
SigmaX	593.93	550.52	330.97	1475.42	
Mean	13.8123	12.8028	7.697	11.4374	
SigmaX2	9009.7827	7798.4116	3208.8895	20017.0838	
Variance	19.1959	17.8624	15.7481	24.5482	
Standard deviation	4.3813	4.2264	3.9684	4.9546	
Standard error	0.6681	0.6445	0.6052	0.4362	
**ANOVA Summary**					
Source	**SS**	**df**	**MS**	**F**	**P**
Treatment	924.2992	2	462.1496	24.32	< 0.0001
Error	1596.5442	84	19.0065		
Ss/BI	621.3243	42			
Total	3142.1677	128			
**Tukey HSD Test**					
HSD[0.05]	2.24				
HSD[0.01]	2.82				
Control vs 200	No significant				
Control vs 1000	P < 0.01				
200 vs 1000	P < 0.01				

Table S2. Statistical analysis of elatol quantification in trichoblasts.

**Data summary**					
			**Samples**		
	**Control**	**200**	**1000**	**Total**	
N	3.0	3.0	3.0	9.0	
SigmaX	0.864354	0.928288	0.452705	2.245347	
Mean	0.288118	0.309429	0.150902	0.249483	
SigmaX2	0.256286	0.292713	0.069368	0.618367	
Variance	0.003625	0.002736	0.000527	0.007274	
Standard deviation	0.06021	0.052311	0.022598	0.085287	
Standard error	0.034762	0.030202	0.013255	0.028429	
**ANOVA Summary**					
Source	**SS**	**df**	**MS**	**F**	**P**
Treatment	0.44414	2	0.022207	10.57	0.025316
Error	0.0084	4	0.0021		
Ss/BI	0.0054	2			
Total	0.058191	8			
**Tukey SD**					
HSD[0.05]	0.13				
HSD[0.01]	0.21				
Control vs 200	No significant				
Control vs 1000	P < 0.01				
200 vs 1000	P < 0.01				

Table S3. Statistical analysis of fouling coverage over algae thalli.

**Data summary**					
			**Samples**		
	**Control**	**200**	**1000**	**Total**	
N	10	10	10	30	
SigmaX	53.28	805.95	924.74	1783.97	
Mean	5.328	80.595	92.474	59.4657	
SigmaX2	740.291	65619.1729	86527.3648	152886.828	
Variance	50.7128	73.737	112.5509	1613.8574	
Standard deviation	7.1213	8.587	10.609	40.1728	
Standard error	2.252	2.7155	3.3549	7.3345	
**ANOVA Summary**					
Source	**SS**	**df**	**MS**	**F**	**P**
Treatment	44668.8575	2	22334.4287	234.28	P < 0.0001
Error	1715.9623	18	95.3312		
Ss/BI	417.0435	9			
Total	46801.8633	29			
**Tukey HSD Test**					
HSD[0.05]	11.17				
HSD[0.01]	14.53				
Control vs 200	P < 0.01				
Control vs 1000	P < 0.01				
200 vs 1000	No significant				

Table S4. Statistical analysis of ABC gene expression.

Gene	Time	logFC	p-value
ABCB	24 h	10.155	2.10^{-4}
ABCB	48 h	10.202	8.10^{-7}
ABCB	72h	1.751	1.0
ABCD	24 h	10.981	8.10^{-7}
ABCD	48 h	6.018	$1.8.10^{-3}$
ABCD	72h	2.171	1.0
ABCF	24 h	9.931	4.10^{-4}
ABCF	48 h	3.988	1.0
ABCF	72h	3.893	1.0
ABCG1	24 h	9.418	6.10^{-4}
ABCG1	48 h	6.997	4.10-2
ABCG1	72h	3.893	1.0
ABCG2	24 h	9.896	3.10^{-4}
ABCG2	48 h	4.186	1.0
ABCG2	72h	-0.162	1.0
ABCG3	24 h	10.061	5.10^{-5}
ABCG3	48 h	5.214	0.2
ABCG3	72h	1.013	1.0

The functional annotation and classification of candidate genes into ABC protein subfamilies was confirmed through SmartBLAST (https://blast.ncbi.nlm.nih.gov/smartblast/), which searches the query against the nr protein database with an optimized version of BLAST targeted to find closely related sequences. SmartBLAST also simultaneously searches the landmark dataset (http://bit.ly/2aOVhPO) with BLASTP and performs a multiple sequence alignment on the six sequences (the query plus up to five matched subject sequences) using the COBALT multiple sequence alignment tool. The cDNA sequences were previously translated into protein sequences by using the Expasy translate tool (https://web.expasy.org/translate/) and standard genetic code.

Table S5. Best hits for *L. dendroidea* gene annotated as ABCB protein (LdABCB) through SmartBLAST.

Description	Max score	Total score	Query cover	E value	Identity	Accession
ABC transporter B family protein [*Dictyostelium discoideum* AX4]	1048	1501	97%	0.0	43.54%	XP_629136.1
ABC transporter B family member 4 isoform X1 [Glycine max]	976	976	97%	0.0	42.73%	XP_003518599.1
ATP-dependent translocase ABCB1 [*Mus musculus*]	945	1738	97%	0.0	40.38%	NP_035205.1
ATP binding cassette subfamily B4 [*Arabidopsis thaliana*]	943	1376	96%	0.0	41.91%	NP_001323911.1
multidrug resistance protein 1 [Danio rerio]	942	942	99%	0.0	40.12%	XP_001922717.3

Table S6. Best hits for *L. dendroidea* gene annotated as ABCD protein (LdABCD) through SmartBLAST.

Description	Max score	Total score	Query cover	E value	Identity	Accession
peroxisomal ABC transporter 1 [*Arabidopsis thaliana*]	724	724	93%	0.0	35.51%	NP_001328232.1
ABC transporter D family member 1-like isoform X2 [Glycine max]	721	721	94%	0.0	35.79%	XP_014631244.1
ATP-binding cassette sub-family D member 1 [*Homo sapiens*]	312	540	84%	3e-90	30.92%	NP_000024.2
Peroxisomal Membrane Protein related [*Caenorhabditis elegans*]	307	531	89%	1e-88	30.25%	NP_001293680.1
ATP-binding cassette sub-family D member 1 isoform X1 [*Danio rerio*]	306	306	43%	8e-88	29.97%	XP_005166771.1

Table S7. Best hits for *L. dendroidea* gene annotated as ABCF protein (LdABCF) through SmartBLAST.

Description	Max score	Total score	Query cover	E value	Identity	Accession
ATP-binding cassette protein subfamily F, member 2, putative [*Leishmania*	219	219	91%	8e-62	28.47%	XP_003860258.1
putative AAA family ATPase Gcn20 [*Schizosaccharomyces pombe*]	217	217	90%	3e-60	29.72%	NP_595837.1
uncharacterized protein Dmel_CG9281, isoform D [*Drosophila*	211	211	88%	7e-59	29.62%	NP_001285284.1
ATP-binding cassette family ATPase ARB1 [*Saccharomyces cerevisiae*	207	207	94%	2e-57	28.23%	NP_010953.1
ABC transporter-related protein [*Dictyostelium discoideum* AX4]	206	206	87%	1e-56	31.53%	XP_637946.1

Table S8. Best hits for *L. dendroidea* gene annotated as ABCG protein (**LdABCG1**) through SmartBLAST.

**Description**	**Max score**	**Total score**	**Query**	**E value**	**Identity**	**Accession**
ATP-binding cassette sub-family G member 4 [*Mus musculus*]	290	290	84%	5e-88	33.52 %	NP_620405.3
ABC-2 type transporter family protein [*Arabidopsis thaliana*]	282	282	82%	1e-85	33.78 %	NP_175734.1
ATP-binding cassette sub-family G member 4 isoform X1 [*Homo sapiens*]	275	275	85%	2e-83	32.40 %	XP_011541254.1
ABC transporter domain-containing protein [*Caenorhabditis elegans*]	274	274	86%	9e-82	32.46 %	NP_001367851.1
ABC transporter G family member 10 [Glycine max]	272	272	83%	1e-81	33.84 %	XP_003539614.1

Table S9. Best hits for *L. dendroidea* gene annotated as ABCG protein (**LdABCG2**) through SmartBLAST.

**Description**	**Max score**	**Total score**	**Query cover**	**E value**	**Identity**	**Accession**
white [*Drosophila melanogaster*]	193	193	36%	9e-50	27.60 %	NP_476787.1
ABC-2 type transporter family protein [*Arabidopsis thaliana*]	191	191	33%	6e-49	28.92 %	NP_191069.2
ATP-binding cassette sub-family G member 2 isoform X1 [*Danio rerio*]	187	187	37%	5e-48	26.71 %	XP_009292498.3
ABC transporter G family protein [*Dictyostelium discoideum* AX4]	179	179	34%	1e-45	27.88 %	XP_646231.1
ABC transporter G family member 20 [Glycine max]	179	179	32%	7e-45	28.34 %	XP_003553533.1

Table S10. Best hits for *L. dendroidea* gene annotated as ABCG protein (**LdABCG3**) through SmartBLAST.

Description	Max score	Total score	Query cover	E value	Identity	Accession
pleiotropic drug resistance 1 [*Arabidopsis thaliana*]	543	702	91%	4e-168	31.19%	NP_566543.1
pleiotropic drug resistance protein 1 [*Glycine max*]	521	521	90%	6e-160	29.79%	XP_003546230.1
ATP-binding cassette sub-family G member 2 isoform X1 [*Danio rerio*]	192	343	76%	6e-50	29.37%	XP_021325521.1
ATP-binding cassette sub-family G member 1 isoform 6 [*Homo sapiens*]	188	188	39%	2e-48	29.55%	NP_997511.1
ATP-binding cassette sub-family G member 3 isoform X2 [*Mus musculus*]	162	162	39%	1e-39	27.96%	XP_017176394.1

Table S11. Results of the incubation assay about the effect of CSA on area of the CC.

Treatment	CC area (µm^2)
Control	8.18
Control	9.06
Control	9.29
Control	16.18
Control	13.26
Control	10.48
Control	13.02
Control	11.41
Control	9.17
Control	14.12
Control	15.7
Control	15.64
Control	15.74
Control	18.81
Control	17.05
Control	19.08
Control	23.8
Control	19.25
Control	10.59
Control	13.6
Control	12.59
Control	9.68
Control	10.4
Control	14.31
Control	16.89
Control	22.32
Control	10.44
Control	13.63
Control	10.17
Control	13.25
Control	9.55
Control	12.45
Control	11.56
Control	10.59
Control	8.69
Control	12.72
Control	9.92
Control	16.16
Control	10.63
Control	8.64
Control	19.08
Control	22.21
Control	24.62

CSA 200 ng/mL	15.49
CSA 200 ng/mL	18.94
CSA 200 ng/mL	20.23
CSA 200 ng/mL	19.69
CSA 200 ng/mL	16.92
CSA 200 ng/mL	11.83
CSA 200 ng/mL	14.79
CSA 200 ng/mL	8.23
CSA 200 ng/mL	7.15
CSA 200 ng/mL	9.06
CSA 200 ng/mL	10.92
CSA 200 ng/mL	14.82
CSA 200 ng/mL	8.15
CSA 200 ng/mL	5.37
CSA 200 ng/mL	15.43
CSA 200 ng/mL	17.6
CSA 200 ng/mL	17.05
CSA 200 ng/mL	7.09
CSA 200 ng/mL	9.38
CSA 200 ng/mL	8.79
CSA 200 ng/mL	10.48
CSA 200 ng/mL	15.13
CSA 200 ng/mL	7.06
CSA 200 ng/mL	11.53
CSA 200 ng/mL	16.6
CSA 200 ng/mL	17.81
CSA 200 ng/mL	15.77
CSA 200 ng/mL	9.69
CSA 200 ng/mL	19.27
CSA 200 ng/mL	10.31
CSA 200 ng/mL	9.43
CSA 200 ng/mL	11.52
CSA 200 ng/mL	10.27
CSA 200 ng/mL	8.94
CSA 200 ng/mL	18.47
CSA 200 ng/mL	9.94
CSA 200 ng/mL	19.81
CSA 200 ng/mL	14.76
CSA 200 ng/mL	15.32
CSA 200 ng/mL	9.18
CSA 200 ng/mL	8.38
CSA 200 ng/mL	11.06
CSA 200 ng/mL	12.86
CSA 1000 ng/mL	12.64
CSA 1000 ng/mL	5.49
CSA 1000 ng/mL	9.13

CSA 1000 ng/mL	3.88
CSA 1000 ng/mL	6.86
CSA 1000 ng/mL	9.33
CSA 1000 ng/mL	4.1
CSA 1000 ng/mL	16.2
CSA 1000 ng/mL	3.69
CSA 1000 ng/mL	7.69
CSA 1000 ng/mL	11.55
CSA 1000 ng/mL	10.83
CSA 1000 ng/mL	9.56
CSA 1000 ng/mL	13.79
CSA 1000 ng/mL	14.48
CSA 1000 ng/mL	9.91
CSA 1000 ng/mL	6.55
CSA 1000 ng/mL	13.65
CSA 1000 ng/mL	8.3
CSA 1000 ng/mL	11.62
CSA 1000 ng/mL	12.85
CSA 1000 ng/mL	10.15
CSA 1000 ng/mL	4.47
CSA 1000 ng/mL	3.26
CSA 1000 ng/mL	7.03
CSA 1000 ng/mL	2.31
CSA 1000 ng/mL	3.78
CSA 1000 ng/mL	4.62
CSA 1000 ng/mL	3.43
CSA 1000 ng/mL	5.27
CSA 1000 ng/mL	9.08
CSA 1000 ng/mL	6.73
CSA 1000 ng/mL	7.35
CSA 1000 ng/mL	3.75
CSA 1000 ng/mL	2.23
CSA 1000 ng/mL	12.34
CSA 1000 ng/mL	9.17
CSA 1000 ng/mL	4.72
CSA 1000 ng/mL	6.03
CSA 1000 ng/mL	2.51
CSA 1000 ng/mL	14.41
CSA 1000 ng/mL	1.82
CSA 1000 ng/mL	4.41

Table S12. Results of the incubation assay about the effect of CSA on elatol content.

Treatment	Elatol concentration
Control	0.335025383591322
Control	0.309105269620527
Control	0.220222887700245
CSA 200 ng/mL	0.365705077776143
CSA 200 ng/mL	0.300298062687688
CSA 200 ng/mL	0.262285249165043
CSA 1000 ng/mL	0.138372276327124
CSA 1000 ng/mL	0.136933988893402
CSA 1000 ng/mL	0.177398648102593

Table S13. Results of the incubation assay about the indirect effect of CSA on fouling.

Treatment	Fouled area (%)
Control	0.67
Control	7.78
Control	24.48
Control	1.32
Control	5.94
Control	2.70
Control	4.48
Control	3.48
Control	0.67
Control	1.76
CSA 200 ng/mL	72.48
CSA 200 ng/mL	100.0
CSA 200 ng/mL	71.67
CSA 200 ng/mL	85.67
CSA 200 ng/mL	79.78
CSA 200 ng/mL	75.00
CSA 200 ng/mL	85.33
CSA 200 ng/mL	79.33
CSA 200 ng/mL	83.33
CSA 200 ng/mL	73.36
CSA 1000 ng/mL	81.33
CSA 1000 ng/mL	66.78
CSA 1000 ng/mL	100.00
CSA 1000 ng/mL	100.00
CSA 1000 ng/mL	93.67
CSA 1000 ng/mL	96.48
CSA 1000 ng/mL	100.00
CSA 1000 ng/mL	96.67
CSA 1000 ng/mL	97.33
CSA 1000 ng/mL	92.48

Figure S1. Multiple alignment of LdABCB (translated sequence) and five best hit protein sequences (SmartBLAST). Alignment columns with gaps are colored grey. The red color indicates highly conserved columns and blue indicates less conserved ones.

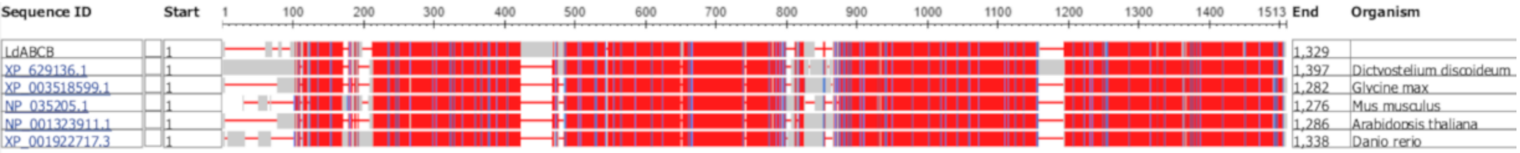

Figure S2. Multiple alignment of LdABCD (translated sequence) and five best hit protein sequences (SmartBLAST). Alignment columns with gaps are colored grey. The red color indicates highly conserved columns and blue indicates less conserved ones.

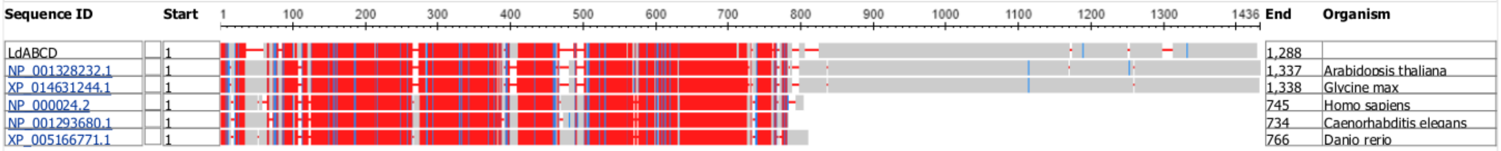

Figure S3. Multiple alignment of LdABCF (translated sequence) and five best hit protein sequences (SmartBLAST). Alignment columns with gaps are colored grey. The red color indicates highly conserved columns and blue indicates less conserved ones.

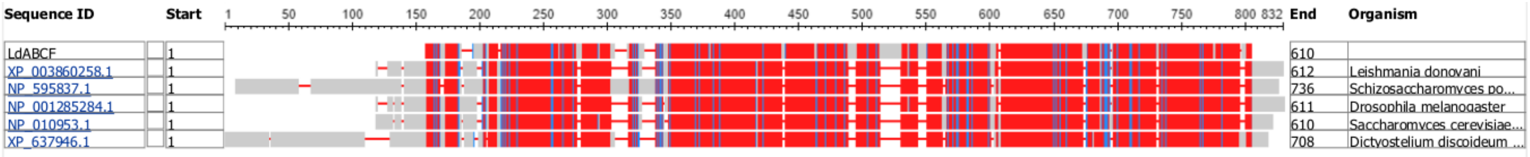

Figure S4. Multiple alignment of LdABCG1 (translated sequence) and five best hit protein sequences (SmartBLAST). Alignment columns with gaps are colored grey. The red color indicates highly conserved columns and blue indicates less conserved ones.

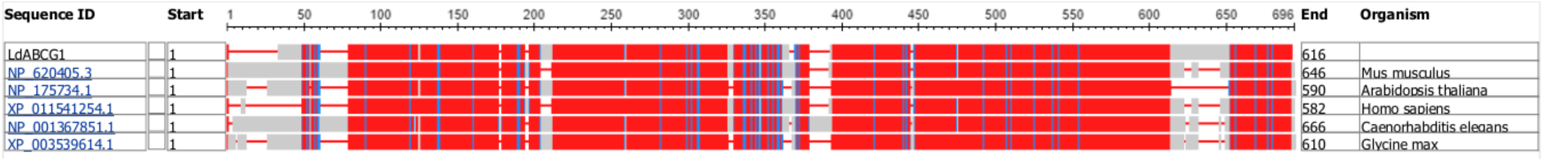

Figure S5. Multiple alignment of LdABCG2 (translated sequence) and five best hit protein sequences (SmartBLAST). Alignment columns with gaps are colored grey. The red color indicates highly conserved columns and blue indicates less conserved ones.

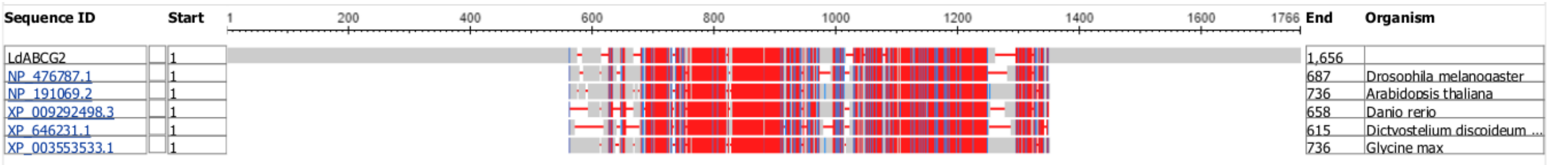

Figure S6. Multiple alignment of LdABCG3 (translated sequence) and five best hit protein sequences (SmartBLAST). Alignment columns with gaps are colored grey. The red color indicates highly conserved columns and blue indicates less conserved ones.

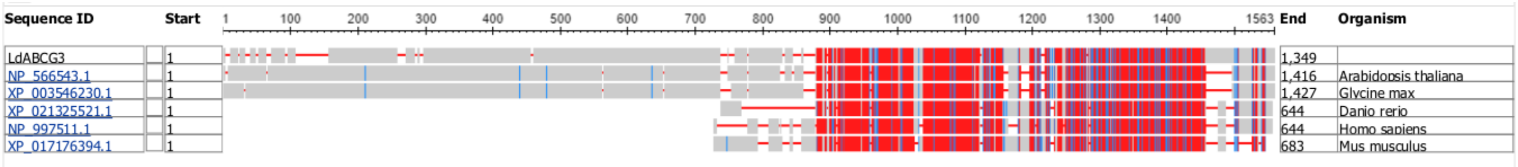

Supplement: Supplementary file 1 [file cells-12-02259-s001.zip › cells-2527759-supplementary.pdf]
